# Long-Term Prognosis after Stroke: A Community-Based Study in Japan

**DOI:** 10.2188/jea.11.8

**Published:** 2007-11-30

**Authors:** Toshiko Ikebe, Hideki Ozawa, Minoru Iida, Takashi Shimamoto, Kyoko Handa, Yoshio Komachi

**Affiliations:** 1 Department of Public Health and Hygiene, Oita Medical University.; 2 Department of Epidemiology and Mass Examination, Osaka Medical Center for Cancer and Cardiovascular Diseases.; 3 Institute of Community Medicine, University of Tsukuba.; 4 Toyama Prefectural Takaoka Health Center.; 5 Osaka Prefectural Institute of Public Health.

**Keywords:** stroke, prognosis, ADL, dependent, community

## Abstract

Stroke is the leading cause of severe disability in the elderly. Under the national insurance for care and assistance for the elderly starting in 2000, data must be obtained on the prognostic status of stroke patients in communities. We identified 322 incident strokes in six communities (total census population= 71,610) during the two- or three-years survey period between 1987 and 1990, and we completed a follow-up of the respective prognoses of most of these patients at one, three, and five years after the onset (n=315 stroke patients) (98%). One year after stroke, 33% of the 315 strokes were dead, 13% were dependent, and 54% were independent. After three years, 44% were dead, 13% were dependent, and 43% were independent. After five years, 52% were dead, 9% were dependent, and 39% were independent. The long-term prognosis was poorer with increased age, and poorer for women than for men except in the case of men ages less than 55 years old at onset. Among patients who were dependents, the proportion of taken care at home was approximately 30% one year after onset, and 50% three to five years after onset. It is estimated that approximately 17 dependents from 127 incident strokes in a population of around 70,000 every year.

Because the average survival time of dependents was about 4 years, the prevalence of dependents is estimated to be 68, indicating that the prevalence is about 10 persons per 10,000.

Over the period of this study, and as compared with the reported proportions in community-based studies in the 1970’s, the proportion of deaths declined and that of independents increased, probably due to reduced severity of stroke. However, the proportion of dependents did not change significantly over time. Thus, under the terms of the new national insurance, it is essential for family and communities to cooperate in taking care of dependent stroke patients.

## INTRODUCTION

Stroke mortality in Japan was much higher than that in other developed countries^1^^)^. In response to high stroke mortality, community-based stroke prevention programs have been carried out in several communities. According to the vital statistics of Japan, stroke mortality declined from 175.8 per 100,000 in 1970 to 96.9 per 100,000 in 1994, comprising 25% of total deaths in 1970, and 14% in 1994^2^^)^. The decline in stroke mortality was due to a decline in stroke incidence and to increased survival rate after stroke, which was attributable to improvements in blood pressure control programs as well as nationwide improvements in lifestyle^1^^,^^3^^-^^5^^)^.

However, the number of stroke patients was 360,000 in 1970, which increased to 1,420,000 in 1993 (3.9 times)^6^^)^, probably due to an increased proportion of the aged among the population and to prolongation of survival after stroke. Based on the influence of these factors, the number of stroke patients is expected to double in the next 15 years^7^^)^. Further, the number of the bedridden elderly, 40 to 60% of whom are bedridden due to stroke, has increased with the progressive aging of Japanese populations^8^^)^. The increasing number of bedridden elderly who need nursing care is one of the most important social problems facing Japan. In an effort to address this problem early on, the Ministry of Health and Welfare launched the “Zero Bed-ridden Elderly Strategy” in 1990^8^^)^. In addition to the increased number of dependent elderly, reduced availability of in-home care due to an increased number of nuclear families has become a potential problem. Therefore, the Ministry of Health and Welfare introduced a program of insurance for care and assistance for the elderly, to be implemented in the year 2000. At this juncture, it is essential to estimate the long-term prognostic status of stroke patients in communities. To establish this estimate, we investigated the survival and the activity of daily living (ADL) for stroke patients at one, three, and five years after the stroke onset in six communities.

## MATERIALS AND METHODS

### Study Populations

The surveyed populations were six communities where follow-ups after stroke were conducted between 1990 and 1991 as a part of the collaborative study on community care system for tertiary prevention of cerebrovascular disease (Chairman: Yoshio Komachi) under the health promotion project for the aged, the Ministry of Health and Welfare^9^^)^.

Five rural rice-farming communities, including Town I in Akita prefecture (census population: n= 6,298), Towns I and K in Ibaraki prefecture (n= 23,547 and 17,217, respectively), Town N in Kochi prefecture (n= 13,965), and Town M in Oita prefecture (n= 5,817), were surveyed, along with a community in the fishing district of City U in Toyama prefecture (n= 4,766).

### Identification of Incident Stroke

We registered the number of strokes occurring in these six communities, two communities were observed for two years between 1987 and 1990, and four others for three years. Stroke was defined as a focal neurological disorder with rapid onset that persisted 24 hours or more, or until death^10^^)^. Thus, transient ischemic attack was not included. To make the registry as complete as possible, information was obtained from five sources: (1) reports by local physicians, (2) medical bills of the national health insurance, (3) death certificates, (4) ambulance records, and (5) records by public health nurses. The completeness of the combined case finding sources was established in Town I in Akita where a single detailed household survey revealed no cases undetected by the system^3^^)^. To confirm stroke diagnosis, our study physicians or public health nurses visited the patients or their families, and interviewed them regarding the relevant events. Hospital records were also reviewed. Lastly, a panel of three to four epidemiologist-physicians made the final diagnosis of stroke according to the standardized criteria^10^^,^^11^^)^. Determination of stroke subtypes (ie, intraparenchymal hemorrhage, subarachnoid hemorrhage, and ischemic stroke) was done primarily by computed tomography (CT) in a standardized way^12^^)^. Strokes diagnosed clinically but showing no lesions on CT were regarded as unclassified-type strokes. Stroke cases without CT films were classified according to the clinical criteria^13^^)^ based on those set out by Millikan^14^^)^.

### Determinant of Survival and ADL for Stroke Patients

Survival and activities of daily living (ADL) among stroke patients at one year after onset have been reported elsewhere^15^^)^. The present study was extended to examine the prognosis of stroke patients at three and five years after onset in six communities. We obtained information on the prognosis of stroke patients through visits or phone calls by epidemiologist-physicians or public health nurses, using the standardized questionnaire.

ADL were classified into four categorical ranks according to the classification of ADL proposed by the Ministry of Health and Welfare^16^^)^. Rank J defined those who were totally or nearly independent when eating, toileting, dressing, and walking outdoors. Rank A defined those who were able to walk indoors independently but not able to go outdoors without help. Rank B defined those who remained in bed almost all day but were able to maintain a sitting position there. Rank C defined those who remained in bed all day and were unable to maintain a sitting position there. We regarded ranks J and A as independent, and ranks B and C as dependent.

Because the prognosis of stroke patients did not vary substantially among communities, we present the data as cumulative.

### Data Analysis

The proportions of deaths and ADL among the surviving patients at one, three, and five years after onset were sorted by sex and age at onset (< 55, 55- 64, 65- 74, and 75+ years old). Kaplan-Meier method was used to examine survival curves according to sex and age groups, and differences in survival rates by sex and age groups was tested using log-rank test. Sex-specific relative risks of death during five years after stroke onset were calculated adjusting for age and stroke subtypes using the Cox proportional hazards model.

In addition, we examined the location (home vs institution) of the stroke dependents at one, three, and five years after onset, stratified by age groups (< 64 vs >= 65 years old). Differences in the proportions between sex and age groups, respectively, were tested using chi-square tests.

## RESULTS

There were 322 incident stroke patients in the surveyed communities during the two- or three-year survey period between 1987 and 1990 (Table 1). The incidence of stroke in these communities was 1.4 to 4.1 per 1,000 persons per year, with 1.85 average. The sex ratio of the 322 patients was about 1:1. The mean age was higher in women (73.2 years) than in men (67.8 years). There were 189 ischemic strokes (59%), 71 intra-parenchymal hemorrhages (22%), 36 subarachnoid hemorrhages (11%), and 26 unclassified strokes (8%). Information on ADL was not available for 2 strokes at one year after onset, 4 strokes at three years after onset, and 7 strokes at five years after onset because these patients moved to communities not included in the survey. The remaining 315 cases were followed up to five years.

**Table 1.** Survey period, population, and the number of strokes in six surveyed areas.

	Survey Period	Census Population in 1990	Number of stroke patients		

			Men	Women	Total
Town I, AKITA	1988 – 1990 (3 years)	6,298	19	14	33
Town I, IBARAKI	1989 – 1990 (2 years)	23,547	30	34	64
Town K, IBARAKI	1989 – 1990 (2 years)	17,217	35	17	52
City U, TOYAMA	1988 – 1990 (3 years)	4,766	17	17	34
Town N, KOCHI	1987 – 1989 (3 years)	13,965	30	37	67
Town M, OITA	1988 – 1990 (3 years)	5,817	34	38	72

Total		71,610	165	157	322

**Figure 1.**
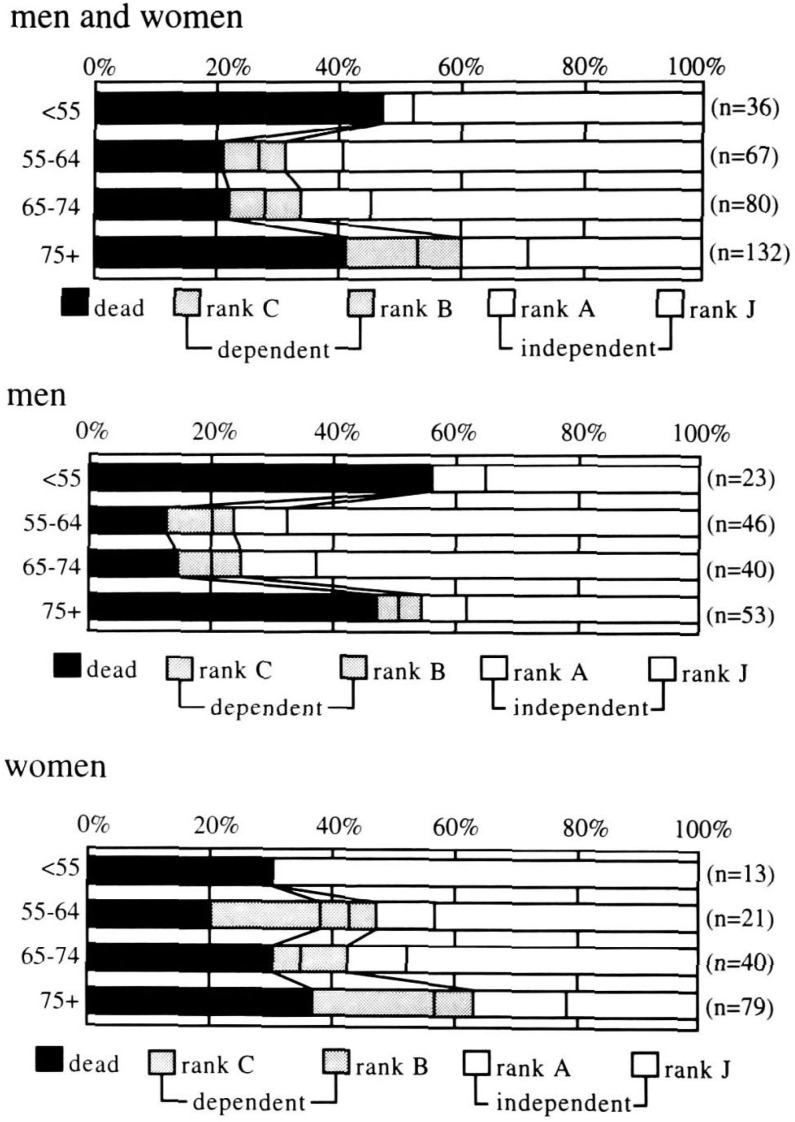
Age-specific prognoses at one-year after onset.

Figure 1 shows the prognosis of 315 strokes at one year after onset by sex and age, respectively. At one year after onset for men and women of all ages combined, 33% (n= 104) of the strokes were dead, 13% (n= 40) were dependent, and 54% (n= 171) were independent. The proportion of death was 47% among patients (men and women) < 55 years of age, 21% in ages 55-64, 23% in ages 65-74, and 42% in ages 75 and older, showing a U shape (differences between < 55 or 75+ and 55-64 or 65-74: p < 0.05). This U-shaped pattern was primarily observed for men; not for women. The prognosis was poorer for women than for men in each age group, except in ages < 55 years, and the difference between sexes was statistically significant in ages 55-64 (p< 0.05).

The proportion of dependents (n= 40) was 0% in ages < 55, 10% in ages 55-64, 11% in ages 65-74, and 18% in ages 75 and older. The proportion of dependents was higher for women than for men in each age group except that of <55, in which group no dependents were observed in either sex, though the sex difference did not reach statistical significance in any age group.

The proportion of independents (n= 171) for men and women inclusively was only 40% in ages 75 and older, while that in other age groups was 53% to 69%. The proportion of independents tended to be lower for women than for men in each age group except that of < 55, in which the opposite trend was observed. However, the sex difference was not statistically significant in any age group.

**Figure 2.**
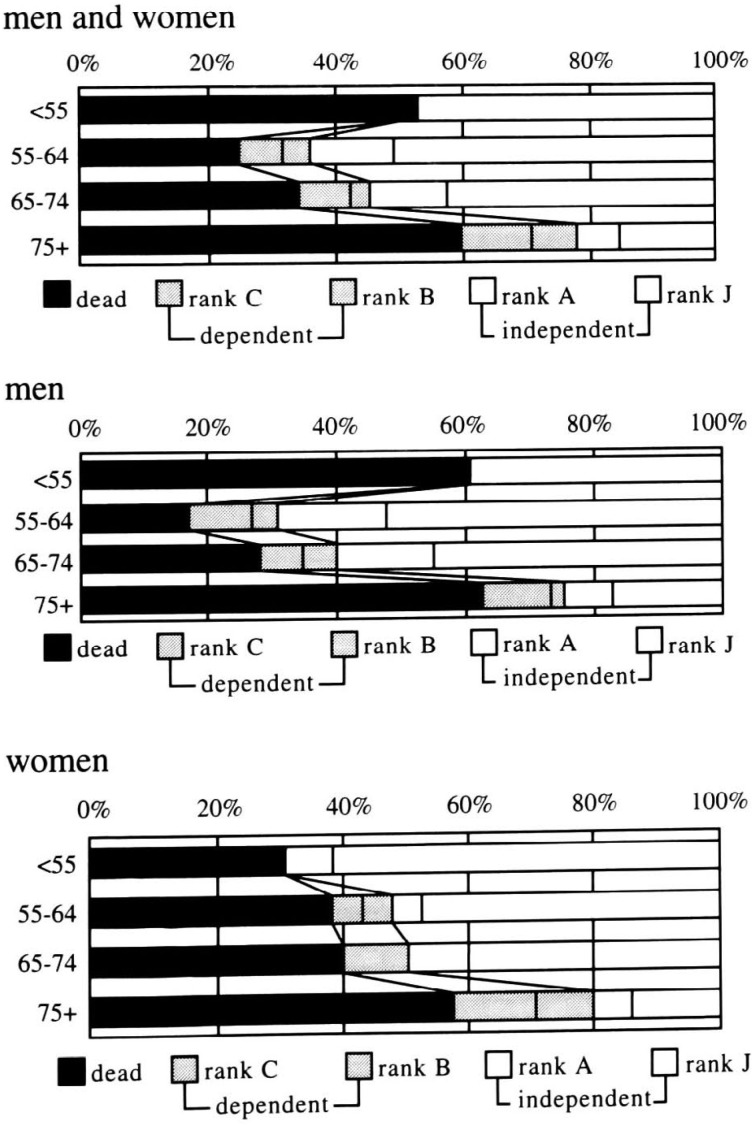
Age-specific prognoses at three-years after onset.

Figure 2 shows the three-year prognoses after stroke by age and sex, respectively. During the one to three years after onset, among the total number of patients, another 35 strokes were dead, bringing the total number of deaths to 139 (44% of total strokes). The proportion of dependents at three years after onset did not change as compared with that one year after onset (13%), while the proportion of independents declined from 54% to 43%. The U-shaped pattern of deaths across the age groups remained for men, though it did not exist for women (differences between < 55 or 75+ and 55-64 or 65-74 : p < 0.05). The prognosis tended to be poorer for women than for men in each age group except that of < 55 years as seen at one year after onset, though the sex difference did not reach statistical significance in any age group.

The proportion of dependents (n= 42) was 0% in ages < 55, 12% in ages 55-64, 11% in ages 65-74, and 19% in ages 75 and older. There was no consistent difference between sexes in the age-specific proportion of dependents. The proportion of independents (n= 134) for men and women inclusively was only 22% in ages 75 and older, while that in other ages was 50% to 64% (p for difference < 0.05). As seen at one year after onset, the proportion of independents tended to be lower for women than for men in each age group except that of < 55, in which group the opposite trend was observed, though the sex difference did not reach statistical significance in any age group.

**Figure 3.**
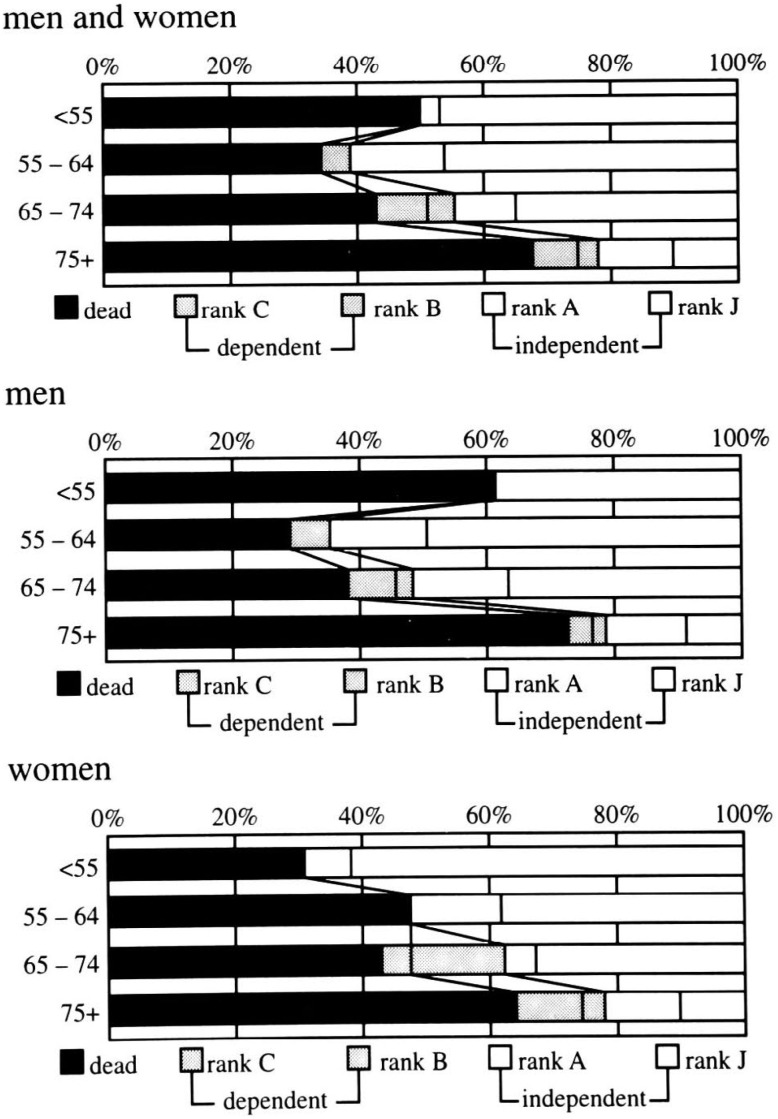
Age-specific prognoses at five-years after onset.

Figure 3 shows the five-year prognoses after stroke by age and sex, respectively. During the three to five years after onset, for the total group of patients, other 25 strokes were dead, bringing the total number of deaths to more than half the total number of strokes (n= 164, 52%). The proportion of dependents declined to 9%, and the proportion of independents declined further to 39%. The U-shaped pattern of deaths across the age groups remained for men, but did not exist for women (differences between < 55 and 55-64, 75+ and 55- 64 or 65-74: p < 0.05). The prognosis tended to be poorer for women than for men in each age group except that of < 55 years as seen at one and three years after onset, though the sex difference did not reach statistical significance in all age groups.

The proportion of dependents (n= 27) was 0% in ages < 55, 4% in ages 55-64, 13% in ages 65-74, and 11% in ages 75 and older. No consistent sex difference was observed in the age-specific proportion of dependents. The proportion of independents (n= 124) for men and women inclusively was only 22% in ages 75 and older while that in other ages 45% to 61% (p for difference < 0.05). As seen at one and three years after onset, the proportion of independents tended to be lower for women than for men in each age group except that of < 55, in which the opposite trend was observed, though the sex difference did not reach statistical significance in any age group.

**Figure 4.**
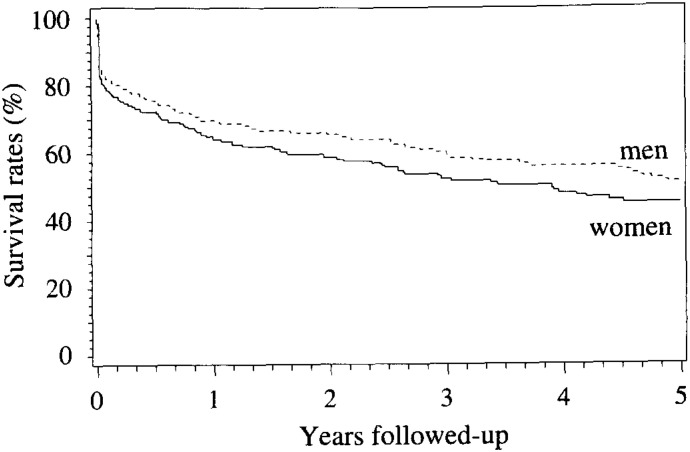
Survival curves among stroke patients by sex. No difference in survival rates was found between men and women: p=0.29.

**Figure 5.**
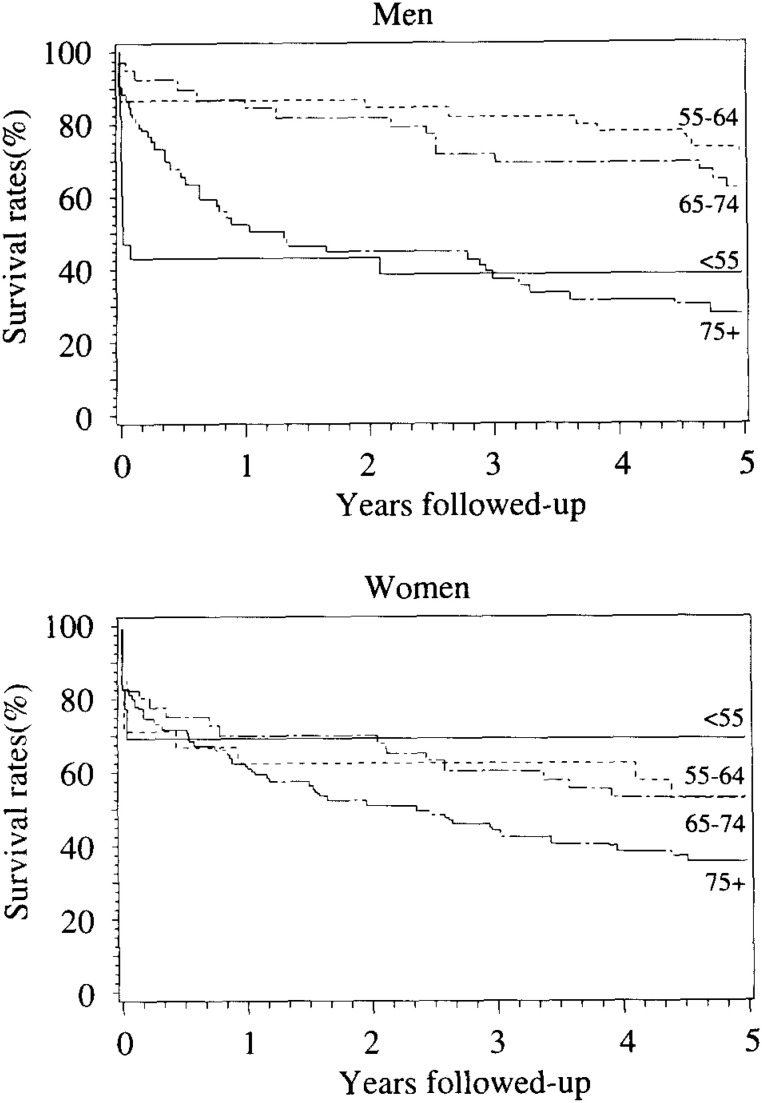
Survival curves among stroke patients by age groups. Defferences in survival rates by age groups : p< 0.001 for men and p= 0.18 for women.

The survival curves comparing prognosis between men and women are shown in Figure 4. Survival rates tended to be lower for women than for men, but the sex difference was not statistically significant. Survival rates varied significantly among age groups for men but not for women (Figure 5). For men, most of deaths (16 cases of total 18 deaths) occurred within one week and few deaths beyond seven days in ages <55. Survival rate five years after onset in ages 55-64 was 66%, that was higher than other age groups. The prognosis was worst in ages 75 and older.

We calculated the sex-specific relative risks and 95% confidence interval (CI) of death adjusting for age. For men, the relative risks compared with ages 55-64 were 3.7 (95%CI, 1.8 to 8.0) for ages <55, 1.3 (0.6 to 2.8) for ages 65-74, and 3.6 (1.9 to 6.8) for ages 75 and older. After further adjustment for stroke subtypes, excess risk for ages <55 was attenuated but remained significant; the relative risks compared with ages 55-64, were 2.9 (1.2 to 7.0) for ages <55, 1.4 (0.7 to 2.9) for ages 65-74, and 3.8 (2.0 to 7.2) for ages 75 and older. For women, the risks of death significantly varied among stroke subtypes but not among age groups. Compared with ischemic stroke, the relative risks were 1.9 (1.1 to 3.4) for intraparenchymal hemorrhage, 1.9 (1.0 to 4.5) for subarachnoid hemorrhage, and 2.1 (1.0 to 4.4) for unclassified strokes.

**Figure 6.**
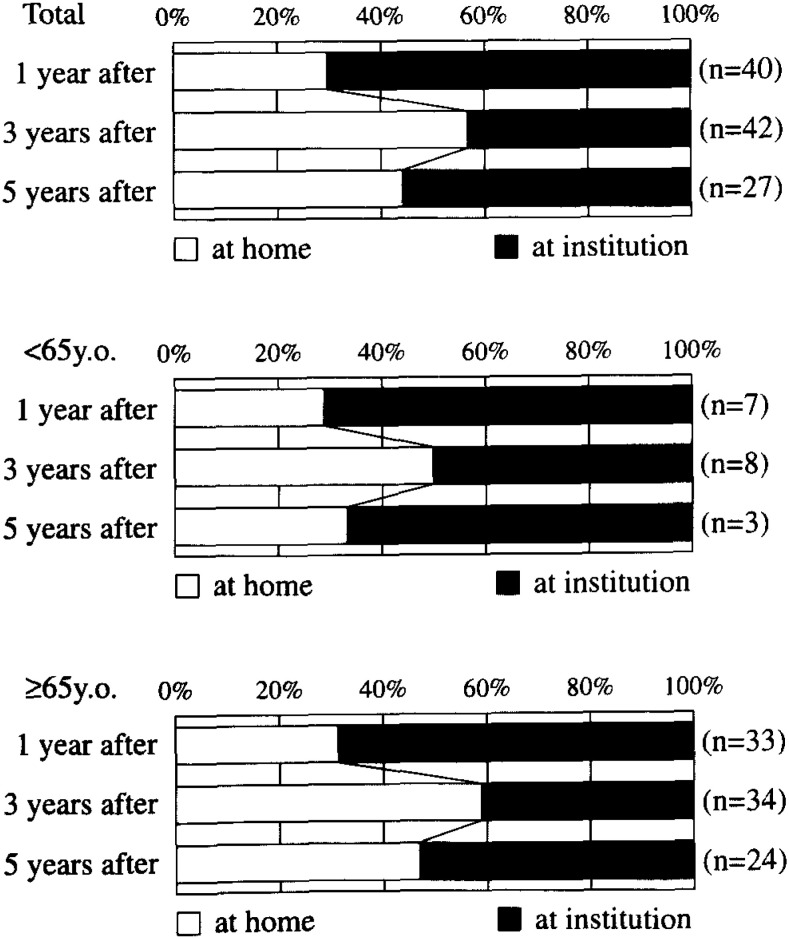
Age-specific locations of the dependent strokes.

Figure 6 shows age-specific locations of the dependent stroke patients at one, three, and five years after onset for men and women inclusively. Because institutions such as hospitals and nursing homes are open to all disabled persons 65 and older, we divided dependents into two age groups, those <65 and those ≥ 65 years. In all age groups, the proportion of dependents cared for at home was 30% after one year at onset, 57% at three years after onset, and 44% at five years after onset. In ages < 65 years, the respective proportions were 29%, 50%, and 50%. In ages ≥ 65 years, the respective proportions were 30%, 59%, and 46%.

## DISCUSSION

In the present community-based study, we successfully examined the long-term prognosis of stroke patients. The proportion of deaths was 33% at one year, 44% at three years, and 52% at five years after onset. The respective proportion of dependents was 13%, 13%, and 9%, and that of independents was 54%, 43%, and 39%. The proportions of deaths and dependents were generally higher among women than among men, partly due to a higher mean age at onset among women than among men. However, survival rates did not differ significantly between men and women according to the life-table analyses.

There was a U-shaped pattern in the proportion of deaths across age groups among men, and higher fatality rates were seen in men < 55 and 75+ than in other age groups, but such a trend was not seen among women. These findings were confirmed by the Cox proportional hazards model adjusting for stroke subtypes. Among 13 deaths of men ages < 55 at one year after onset, 12 (92%) of these deaths occurred within one week of stroke. The 13 deaths were classified as 6 intraparenchymal hemorrhages, 5 subarachnoid hemorrhages, no ischemic strokes, and 2 unclassified strokes. Among 25 deaths of men ages 75+ at one year after onset, 6 (24%) of these deaths occurred within one week of stroke. The 25 deaths were classified as 6 intraparenchymal hemorrhages, no subarachnoid hemorrhages, 16 ischemic strokes, and 3 unclassified strokes. Thus, the higher case-fatality rate within one week of stroke among men ages < 55 was in part due to the high proportion of hemorrhagic strokes. The multivariate analyses supported the findings; the relative risk of death at ages <55 vs 55-64 was 3.7 (95%CI, 1.8 to 8.0), but attenuated to 2.9 (1.2 to 7.0) after adjustment for stroke subtypes. The survivors among men ages < 55 at onset, however, were mostly independent even at three and five years after onset. In older age groups in men, the proportion of deaths increased and that of independents declined gradually from one to five years after onset.

The case-fatality rates in the present study were similar to those reported in a well-designed community-based registry in England, under the auspice of the Oxfordshire Project: 31% within one year, 45% within three years, and 50% within five years^17^^)^. However, the case-fatality rates reported in hospital-based studies^18^^-^^23^^)^ were generally high within three years compared with those in the present study. The high case-fatality rates in hospital-based studies were probably due to non-hospitalization of some strokes with mild signs and symptoms.

In Town I, Akita prefecture, the case-fatality rate of strokes occurring during 1975-1981 was 32% (31/97) within one year, 51% (49/97) within three years, and 61% (59/97) within five years^24^^)^. In the present study, the corresponding rate among strokes occurring during 1987-1990 was 30% (10/33) within one year, 33% (11/33) within three years, and 36% (12/33) within five years. Reduced case-fatality rate between 1975-81 and 1987-90 (p= 0.96 after one year, p= 0.13 after three years, and p= 0.02 after five years) was similarly observed for both sexes, and was probably due to the decreased incidence of severe stroke cases^25^^,^^26^^)^, which may in part be attributable to an intensive hypertension control program^11^^)^. Among the strokes occurring during 1975-1981, the proportion of independents five years after onset was 37% (21/57) for men and 13% (5/40) for women^24^^)^. In the present study, the corresponding proportion of strokes occurring during 1987-1990 was 44% (72/162) for men and 32% (52/153) for women, indicating an increased proportion of independents between 1975-81 and 1987-90 (p = 0.39 for men and p= 0.01 for women). The increased proportion of independents was also probably due to the decreased incidence of severe stroke cases.

The proportion of dependents at five years after onset among strokes occurring during 1975-1981 was 14% (8/57) for men and 10% (5/40) for women^24^^)^. The corresponding proportion among strokes occurring during 1987-1990 was 6% (10/162) for men and 10% (17/153) for women. The proportion of dependents did not change significantly over time.

Similar time trends in the prognoses of stroke patients were reported from Town N in Kochi prefecture, one of the communities surveyed in the present study. Between 1969-78 and 1979-88, the proportion of deaths 3 years after stroke declined, and that of independents increased, while that of dependents did not change^27^^)^.

In the present study, 322 incident strokes occurred in a population of 70,000 during the two- or three-year survey period. Therefore, the average number of incident strokes per year was 127. Because the proportion of dependents at one year after onset was 13%, the estimated number of dependents was 17. Over half of dependents at one year after onset (60%) were dead after five years, which means that the average survival time for dependents was around four years. Therefore, the prevalence of dependents is estimated to be 68 (17×4 year), indicating that the prevalence per 10,000 persons is 10. This estimated prevalence rate is similar to those reported from Town I in Akita prefecture (14 per 10,000)^28^^)^ and Town K in Ibaraki prefecture (12 per 10,000)^29^^)^.

In conclusion, the present community-based study on the long-term prognosis of strokes showed that 10% of incident strokes were dependent at one to five years after onset, and that half of these dependents were taken care at home, the remaining half at institutions. Caring for dependents at home places a physical and mental burden on the family, while caring for dependents at institutions creates a financial burden on a community. Ideally, these burdens can be appropriately addressed under the terms of the national insurance for care and assistance for the elderly.
